# Microbial cell-free protein synthesis and its progression toward industrial use

**DOI:** 10.1099/mic.0.001757

**Published:** 2026-08-12

**Authors:** Rochelle Aw

**Affiliations:** 1 School of Life Sciences, University of Nottingham, Nottingham, NG7 2RD, UK

**Keywords:** cell-free systems, decentralized manufacturing, microbial cell-free protein synthesis

## Abstract

Industrial adoption of microbial cell-free protein synthesis (CFPS), an *in vitro* protein production platform that uses transcription and translation machinery from lysed cells, remains limited despite sustained technical progress across the field. This perspective positions microbial CFPS within a manufacturing context, focusing on its current capabilities, the limits of those capabilities and the concrete steps required for industrial adoption. In CFPS, the absence of cell growth constraints supports rapid protein production, simplified upstream workflows and direct control over reaction conditions, while also lending itself to formats such as lyophilized reagents for storage and transport. Beyond basic protein expression, microbial CFPS is increasingly used as an adaptable production environment in which reaction composition can be tuned, accessory enzymatic functions can be introduced and post-translational modification strategies can be engineered to better align with product requirements. The expanding range of microbial chassis further broadens the design space, allowing platform choice to be guided by functional, regulatory and deployment considerations rather than yield alone. However, increased capability has not yet translated into routine manufacturing use, and persistent barriers include reproducibility, vulnerable supply chains, limited scale-up practice, downstream purification suitable for therapeutic products and the absence of CFPS-specific quality and regulatory expectations. Overall, microbial CFPS is best evaluated not as a replacement for existing biomanufacturing but as a complementary manufacturing paradigm whose industrial relevance will depend on establishing standardization, economics and regulatory readiness.

## Biomanufacturing stress test in the pandemic

The world’s current manufacturing strategies and pipelines were stretched during the severe acute respiratory syndrome coronavirus 2 (SARS-CoV-2) pandemic, limiting production of vaccines and therapeutics [1], and many governments highlighted the need to future-proof biotherapeutic manufacturing to enable a faster response [2]. The limited number of centralized manufacturing facilities meant that even with rapidly developed therapeutics such as mRNA vaccines [3], infrastructure constraints limited production capacity and disrupted existing medication supply. Cold-chain requirements further restricted global distribution, particularly in low-resource environments [4, 5]. Six years on from the SARS-CoV-2 pandemic, despite substantial investment in pandemic preparedness, questions still remain regarding manufacturing capabilities [6].

**Fig. 1.**
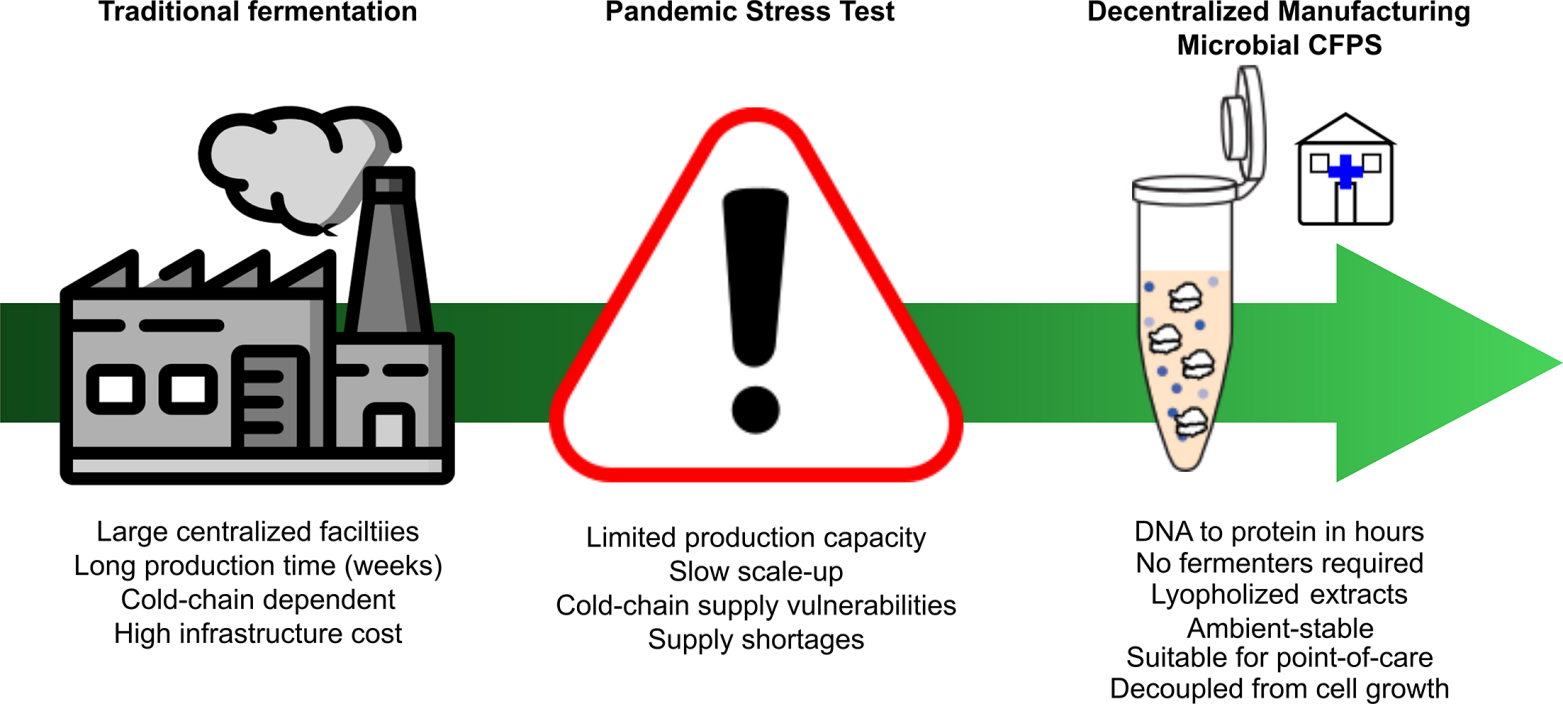
Transition from centralized fermentation to decentralized microbial CFPS. Traditional fermentation and its reliance on centralized infrastructure contributed to bottlenecks during the pandemic. Microbial CFPS provides a rapid, growth-independent, ambient-stable platform for decentralized production.

One way to address these concerns is to move away from centralized manufacturing facilities and look to other technologies that can disrupt our current manufacturing paradigms. This perspective examines how microbial cell-free systems can address these manufacturing limitations. The UK became the first country to introduce a legal framework for point-of-care manufacturing in July 2025 [7]. This framework opens the door to on-demand protein production and potentially even personalized medicine. But achieving decentralized manufacturing requires technologies that go beyond traditional manufacturing techniques, and cell-free protein synthesis (CFPS) offers a platform capable of filling this gap (Fig. 1). CFPS utilizes cellular machinery from lysed cells to power *in vitro* transcription and translation reactions to produce recombinant proteins. While CFPS has been used since the 1960s, where it was first used to help solve the genetic code [8], it has only been in the last 20 years that a resurgence has led to improved production for biotherapeutics [9, 10]. This perspective argues that microbial CFPS should be evaluated not as a replacement for fermentation, but as a distinct manufacturing paradigm optimized for decentralized, rapid-response production.

## Foundations of microbial CFPS

CFPS works on the premise of decoupling cell growth from protein production, and this is achieved using cell lysates. Compared to *in vivo* protein expression, production is rapid and can be completed in a matter of hours, with no specialized equipment required. The reactions are also open and tuneable, and therefore, it is possible to modify reaction conditions far more easily than in growing cells, without concerns over effects on cell growth. Additionally, this decoupling of growth with protein production means that it is possible to produce toxic products in these systems with no repercussions on cell viability. When using CFPS, there is limited cloning or upstream processing such as strain selection, which enables rapid prototyping of variants [11]. Microbial cell-free systems combine high yield potential with low-cost extract preparation, short doubling times and globally established supply chains, making them well suited to decentralized manufacturing contexts.

The fundamental aspect of CFPS is the preparation of the lysates and, once processed, these can be flash frozen at −80 °C. Research has been undertaken to stably lyophilize extracts allowing for a just-add-water system, thereby overcoming cold-chain limitations that have hampered vaccine equity in developing countries [12–15]. However, CFPS requires more than just active cell lysates; an often-criticized aspect is the need to add additional components to enable *in vitro* transcription translation. Depending on the system, these components can be costly and limit the financial viability of using CFPS for manufacturing purposes [16]. In particular, reagents such as DNA templates, nucleoside triphosphates (NTPs) and transcription machinery (e.g. T7 RNA polymerase) can represent significant contributors to overall reaction cost. However, if the focus becomes on decentralized manufacturing or point-of-care models, the implications of cost may be diminished in favour of a reduction in the reliance on large, specialized equipment and facilities. Beyond protein production [17], CFPS reactions are used as biosensors [18–21], for metabolic engineering [22–24] and to produce synthetic cells [25].

Theoretically, any organism can be used to create cell-free lysates [26]; this spans from non-microbial organisms such as rabbit reticulocyte lysates [27], human embryonic kidney cells [28], Chinese hamster ovary (CHO) cells [29, 30] and insect cells [31, 32] to plant cells with the popular tobacco BY-2 platform [33] and to microbial cells such as yeast [34, 35] and bacterial systems [36–39]. As with *in vivo* production platforms, there are advantages and disadvantages of all these systems, and the choice in extract will be informed by the proposed purpose. Eukaryotic systems are often better for post-translational modifications (PTMs) but have limitations in yields and are still costly to produce, whereas bacterial systems often have higher yield but may require more engineering to achieve relevant PTMs for biomanufacturing. Microbial CFPS offers one of the most practical routes toward decentralized manufacturing, owing to its speed, flexibility and chassis diversity. Without microbial CFPS at its centre, it is difficult to see how decentralized manufacturing could meet practical performance and cost targets.

## The diversity of microbial cell-free platforms

**Fig. 2.**
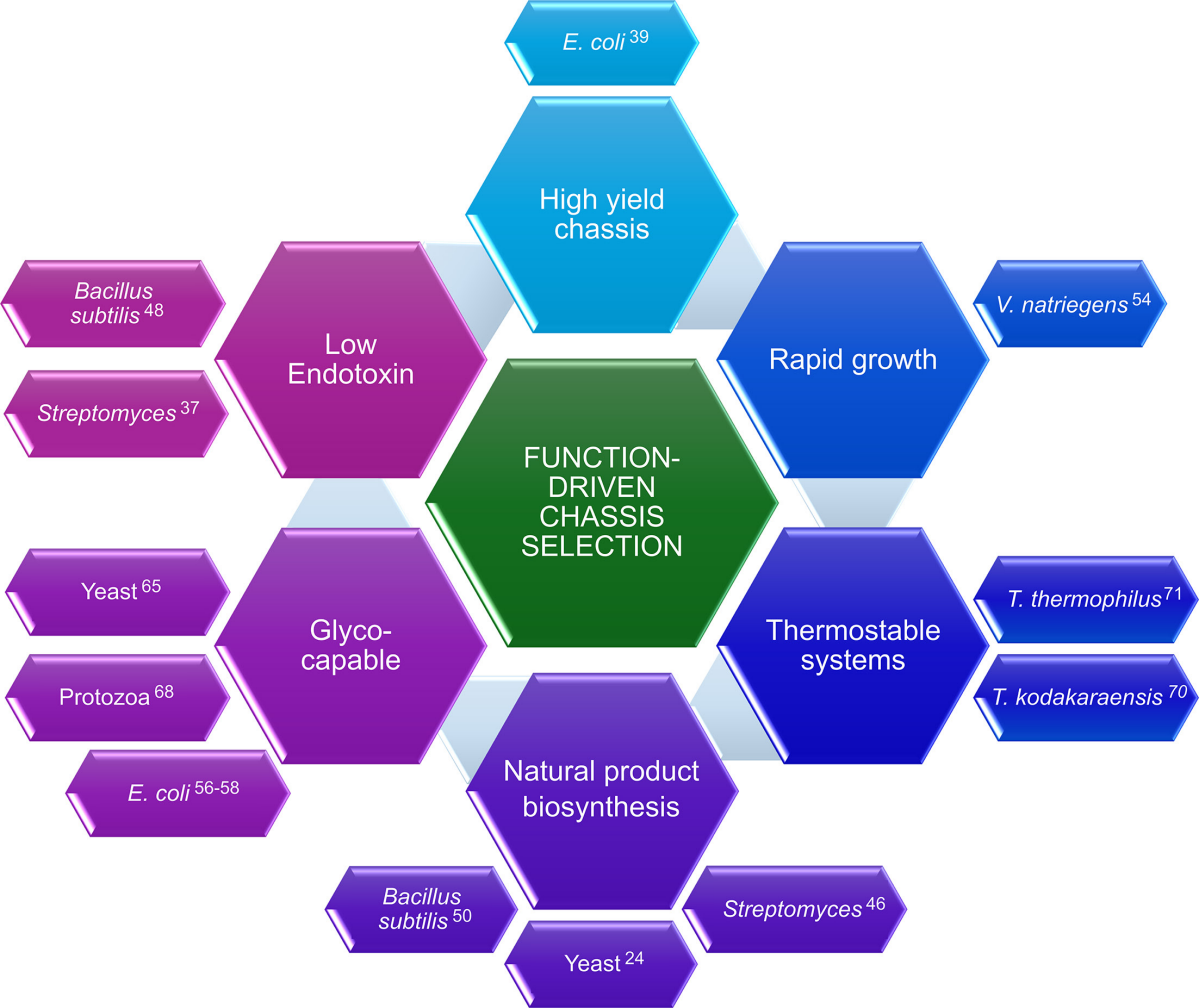
Function-driven chassis selection. Schematic illustrating the selection of microbial chassis based on desired functional attributes rather than taxonomic classification. Key performance characteristics include high protein yield, rapid growth, low endotoxin content, thermostability, glycosylation capability and suitability for natural product biosynthesis. Representative microbial hosts that have been developed for CFPS and exemplify each functional category are shown. Some organisms appear in multiple categories, reflecting the diversity of applications that can be supported by a single chassis. Numbers indicate references describing the corresponding systems.

Microbial lysates provide different functional advantages, with different lysates suited to distinct manufacturing requirements rather than a single universal option (Fig. 2). Chassis selection should be guided by manufacturing requirements, as it is unlikely that even from a CFPS perspective a single platform will be universally optimal. The most reported system is the *Escherichia coli* CFPS lysate, and no fewer than eight commercial platforms are available including from major suppliers such as Promega and NEB [40]. This accessibility has increased the adoption of CFPS, and educational kits also mean that exposure to this technology is becoming more broadly available [41–43]. Production yields have been reported of up to 4 g l^−1^ [44], and the cost per gram of protein has recently dropped over 94% to ~USD 39/g_protein_ compared to the standard $4083/g_protein_ for the commonly used PANOx-SP system [16].

Potentially due to the success of the *E. coli* CFPS platform, no other bacterial CFPS kits are available commercially. However, other bacterial lysates have been developed, and uptake of non-*E. coli* systems is most likely where specific functional advantages, such as specialized biosynthetic pathways [45] or the production of natural products [46], justify reduced yields. Many excellent reviews cover the plethora of bacterial cell-free expression platforms [47], illustrating the potential need to move beyond the standard *E. coli* platform despite its superior yield. *Bacillus subtilis*, which was adapted for CFPS in 1979 [48], has since been used to evaluate prototyping of regulatory units [49] and natural product biosynthesis [50]. As a Gram-positive bacterium, endotoxin concerns are reduced, and its reduced codon bias lowers the need for codon optimization in many cases [51]. Another Gram-positive bacterium, *Corynebacterium glutamicum*, has further advantages such as a lack of proteases [52]. *Vibrio natriegens* grows rapidly and, as a result, has a high concentration of ribosomes to support this growth [53], an advantageous aspect to maximize cellular content contributing to yields of up to 1.6 g l^−1^ when coupled with the T7 RNA polymerase [54]. Furthermore, lysates derived from this organism exhibit low endotoxin levels and do not benefit from additional codon optimization of recombinant genes in CFPS reactions, likely due to their broad tRNA pool, which supports high translation capacity [52].

Yet to be realistic about the production of more complex proteins, it becomes essential to look beyond bacterial systems, especially when thinking about PTMs. While the open reaction environment means that it is possible to modify the reaction conditions to produce disulphide bonds, including antibodies [55] with bacterial lysates, when considering glycosylation or phosphorylation, things are more challenging. That is not to say glycosylation cannot be achieved with bacterial lysates [56–58], but this tends to focus on bacterial glycans for conjugate vaccines and more complex or branched glycans fall short and require substantial modifications [59]. It will be necessary for researchers to take advantage of the breadth of species that exist to identify organisms that are suitable for specific products.

An obvious deviation away from bacterial lysates is to fungal lysates. Part of the draw of a fungal cell-free system is the low cost of producing the lysate. The first fungal extract was *Saccharomyces cerevisiae* in the 1970s [60, 61], but even extensive research has resulted in limited yields reaching a maximum of 12 µg ml^−1^ [34, 62]. *Komagataella phaffii* (syn. *Pichia pastoris*) has been developed that has been reported to have tenfold higher yields of over 100 µg ml^−1^ [35, 63, 64]. This Crabtree-negative yeast can grow to high cell densities due to a lack of detrimental by-product formation such as ethanol, and as a model organism, it is easy to genetically engineer to boost yields further including engineering for improved ribosome content [35]. Further research has investigated alternative energy systems to drive down the cost associated with the energy regeneration using creatine phosphate [65, 66]. Friedrich *et al*. also screened 22 different filamentous fungi, of which 12 showed activity, to create novel CFPS platforms and used these to produce unspecific peroxygenases [67].

Beyond bacteria and yeast, microbial eukaryotes (protozoa) have also been used for cell lysates. An *in vitro* translation system from *Trypanosoma brucei* has been shown to be capable of achieving glycosylation [68], although a coupled *in vitro* transcription translation system has yet to be reported. More extensively, *Leishmania major* CFPS has been developed in a lyophilized format and applied as a biosensor [12, 69].

Researchers still need to think beyond the limitations of standard lysates. For example, a *Thermococcus kodakaraensis* lysate operates at temperatures between 40 and 80 °C, enabling the production of thermostable enzymes that retain activity only under high-temperature conditions [70]. Additionally, a thermostable lysate from *Thermus thermophilus* was also developed and shown to be compatible with microfluidic droplets, showed improved reaction longevity and supported enzyme assay production [71]. It is easy to fall into the trap that, as the most established lysate, *E. coli* is a one-size-fits-all lysate. Nevertheless, fundamentally, the advantages of microbial CFPS platforms still outweigh mammalian and insect cell-free systems from the perspective of cost, growth rate, robustness and improvements in PTMs. Collectively, this diversity highlights that no single microbial CFPS platform will address all manufacturing needs; instead, chassis selection should be driven by the specific regulatory, functional and logistical requirements of the intended application.

## Expanding post-translational modification capabilities in microbial CFPS

Broadly, bacterial CFPS systems are often perceived as limited in post-translational modifications (PTMs), particularly glycosylation, despite increasing evidence that several PTMs can be engineered *in vitro*. Firstly, an advantage of CFPS is the ability to readily modulate the reaction environment to enhance PTMs, for example, through the addition of chaperones that promote disulphide bond formation. Sutro Biopharma has shown the strength of this through developing full-length antibodies in an *E. coli* CFPS platform including genome engineering of the host strain to overexpress chaperones [55, 72]. Additionally, both phosphorylation [73] and prenylation [74] have been achieved using *E. coli* CFPS.

Where things get more interesting is in relation to glycosylation, and it is the lack of natural glycosylation systems in *E. coli* that enable precision glycoengineering *in vitro*. The identification of bacterial oligosaccharyltransferases (OSTs) that can en-bloc transfer glycans to sequence-specific amino acids has expanded the possibility of *in vitro* glycosylation using biological systems instead of chemical synthesis [56–58]. When considering the issues with non-homogenous glycosylation with regard to *in vivo* production, these systems could open new avenues for biomanufacturing. It is the expansion into PTMs using CFPS platforms that will encourage users to move beyond *E. coli* lysates. There have been limited examples of transferring more complex glycans that mimic human-like glycans and are preferentially used for manufacturing, such as GlcNAc_2_Man_3_ using bacterial oligosaccharides, but efficiency is low [56]. Comparatively, eukaryotic systems leverage the native glycosylation machinery instead of heterologous OSTs. While glycosylation in CFPS systems is predominantly explored in non-microbial eukaryotic systems [75], multiple yeast species have been shown to be successful [76, 77]. This often involves the supplementation of microsomes, which can be challenging, and efficiencies still need to be honed.

## Emerging technologies shaping microbial CFPS

What cell-free does well is evidenced by the myriad of excellent research papers [9, 47], but beyond the standard use of biosensors, biomanufacturing, biocatalysis and metabolic engineering, there are now opportunities for novel applications. The open reaction environment of CFPS means that more complex proteins are capable of being formed correctly including virus-like particles (VLPs) [78–80] and adeno-associated virus (AAV) particles [81]. More recently, VLPs that are adapted to act as drug delivery platforms have been developed in CFPS [82]. Coupled to this, the ease of incorporating non-canonical amino acids has opened entirely new avenues for biotherapeutics [83–85]. Vaxcyte has produced a 31-serotype *Streptococcus pneumoniae* vaccine only possible using non-canonical amino acids to attach the antigens to a CFPS-produced carrier protein [86]. While the antigens are still produced *in vivo*, the specificity of the non-canonical amino acid conjugation has resulted in products with high site occupancy.

It is impossible to ignore the impact that machine learning (ML) and artificial intelligence (AI) have had on the world. While it is easy to use these tools and technologies to develop a plethora of designs, wet-lab work needs to be able to match this output [87]. This is one area that CFPS can outcompete *in vivo* applications. Coupling ML and cell-free testing shows the power of rapid testing; Landwehr *et al*. evaluated 10,953 unique reactions to make 9 small molecule pharmaceuticals [23]. DropAI has shown the possibility of screening 100,000 picolitre scale reactions designed using AI, though this was predominantly for system optimization [88]. Gingko Bioworks incorporated ChatGPT-5 to optimize cost and titre of cell-free yields but explicitly had to feed in supplementary data from similar research to generate meaningful outputs [89]. A recent commentary has gone so far as to suggest that combining ML/AI with CFPS may redefine the infamous design-build-test-learn cycle to instead be learn-design-build-test [90].

From a decentralized manufacturing perspective, no field considers these implications more than astropharmacy, where researchers are evaluating using lightweight freeze-dried cell-free lysates to overcome limitations in transporting medicines for space missions [91, 92]. Research into the functionality of cell-free reactions in stimulated microgravity [93], as well as on the international space station, has shown the feasibility of this technology [94]. However, additional challenges arise as lyophilized lysates must also be stable for the length of a journey to Mars, for instance, and purification in resource-limited settings must be addressed.

Together, these advances suggest that microbial CFPS is moving beyond simple protein expression toward an integrated platform linking design and testing. The capacity to assemble VLPs, incorporate non-canonical amino acids and interface with AI-driven design workflows is beginning to alter how individual steps in biomanufacturing can be organized. Instead of multi-week fermentation cycles followed by extensive purification and characterization, microbial CFPS offers a pathway toward high-resolution, iterative manufacturing where design and production occur in the same physical workflow.

These advances preserve the key advantages of microbial systems while avoiding the limitations imposed by maintaining live cultures. This means microbial extracts can be modified and extended in ways that would not be feasible in growing cells. Overall, these changes in how proteins can be designed and produced position microbial CFPS as a credible alternative to established manufacturing routes.

## Barriers to industrial adoption

While these improvements in the platform across multiple species are of great scientific importance, this alone does not necessarily equate to successful industrial processes. Even before thinking of industrial scale, there are implementation problems at lab scale that need to be overcome. Due to the complexity of CFPS, it is evident that reproducibility remains a problem; consistency is difficult to achieve between lab members let alone different research groups [95]. While research has predominantly focused on *E. coli*, increasing system complexity, particularly in eukaryotic CFPS platforms, is likely to exacerbate reproducibility challenges.

To transition to industrial applications, scale of reactions must be considered. Zawada *et al*. highlighted that the system scales linearly up to 100 L reaction volumes [96], but whether despite this or because of it, few groups find it necessary to show application beyond the millilitre scale. Rezvani *et al*. demonstrated expression of a T7 RNA polymerase at the 1 L scale using CFPS [17], but other large-scale expressions are alluded to but not reported explicitly. In part, this limitation is imposed by the price of the reactions, though hopefully implementation of cheaper formulations will soon reduce this particular bottleneck [16]. Improvements in the production of lysates at scale have shown the consistency required to make the steps necessary for large-scale CFPS reactions, which will enable broader adoption of large-scale reactions [97, 98].

From a manufacturing point of view, while lysate can be stockpiled, it does not necessarily correlate to stable supply chains. In fact, stable supply of T7 RNA polymerase, the driver of *in vitro* transcription within the CFPS reaction, was one of the significant challenges of the coronavirus disease 2019 pandemic [99]. Because many CFPS platforms rely on the same transcriptional enzyme, widespread adoption risks exacerbating this bottleneck rather than alleviating it. Alternatives include the use of different promoter–polymerase pairs to reduce reliance on T7 RNA polymerase [100, 101], or the establishment of a closed-loop approach in which T7 RNA polymerase is produced *in situ* as part of the CFPS reaction itself [17]. Beyond T7 RNA polymerase, CFPS platforms remain dependent on multiple biochemically specialized inputs including high-purity NTPs, energy regeneration substrates, translation cofactors and, for eukaryotic systems, mRNA processing enzymes, many of which share supply chains with diagnostics and vaccine manufacturing and therefore represent structural vulnerabilities during periods of global demand.

Building on earlier advances in ML–driven CFPS design and screening, the integration of these approaches with laboratory automation is now enabling fully closed-loop, self-driving workflows. Recent advances in laboratory automation, including benchtop robotics [89, 102] and emerging self-driving CFPS workflows [103], are beginning to transform experimental throughput and reproducibility. These platforms combine ML–guided experimental design with automated execution in a closed-loop workflow and have been demonstrated to optimize CFPS reaction conditions at scale, enabling rapid iteration and reducing operator intervention. Such approaches are particularly attractive for decentralized manufacturing, where minimizing user input and ensuring reproducibility are critical.

Producing proteins is one thing, but decentralized manufacturing also requires purification solutions suitable for therapeutic use. For applications such as metabolic engineering and pathway design, this step can be omitted as CFPS reactions can be used directly, reducing processing time and complexity. However, from a therapeutic production standpoint, purification remains essential to ensure product safety and regulatory compliance. An all-in-one device, coupling CFPS and protein purification, aptly named MANGO (MANufacturing on the GO), starts to tackle the challenges necessary to deploy these systems to low-resource environments [104]. However, the proteins produced are not yet therapeutically applicable, and when combined with the field’s current reproducibility limitations, personalized medicine remains a longer-term rather than near-term ambition. Greater use of laboratory automation with downstream purification technologies will be essential, with self-driving CFPS workflows and modular purification systems together offering a pathway to improved reproducibility, reduced operator burden and ultimately deployable decentralized manufacturing pipelines.

It would be remiss not to mention the issues around biosafety with techniques such as cell-free systems. Kits like BioBits™ lower the technological barrier by increasing general access to the technology and create additional regulatory challenges. If people have access to DNA templates, a component that is already controlled by companies but with the potential to find loopholes [105], they could potentially be making hazardous materials in their own home [106]. Suggestions for combating this at the DNA level have already been implemented including screening for homology to pathogen components, but taking this to the protein level will require a more sophisticated approach that will need to be considered [107]. Building public confidence will require clear quality-control frameworks that demonstrate CFPS-derived products are consistent, traceable and manufactured safely when produced in non-traditional manufacturing facilities. This extends to the consideration of decentralized biomanufacturing, the use of multiple facilities complicates issues such as regulatory approval and steps will need to be taken to ensure safety processes are consistent across all sites.

Overall, these bottlenecks underscore a central tension: while CFPS enables unprecedented flexibility, it demands a level of operational control that the field has not yet standardized. Industry is accustomed to microbial fermentation processes that have been optimized, codified, automated and refined over decades. By contrast, CFPS remains a highly variable research method with extract-to-extract differences, reagent supply dependencies, DNA template variability and batch inconsistencies that make regulated production inherently risky [95, 108].

Until CFPS platforms achieve the reproducibility, quality metrics and supply-chain stability expected of industrial processes, manufacturers will remain cautious. This hesitation is not a failure of imagination; it reflects the absence of shared standards, reference lysates and validated workflows. Bridging this gap will determine whether microbial CFPS stays in the academic domain or progresses into a credible manufacturing technology.

## CFPS and the repositioning of biomanufacturing

The global market size of CFPS is projected to be worth USD 716.26 million by 2034 [109], showing the increasing interest in this field. Sutro Biopharma pioneered the first therapeutic employing CFPS-derived intermediates submitted to the Food and Drug Administration (FDA) and subsequently announced a research collaboration with the FDA aimed at advancing regulatory standards for such products [110, 111]. However, to date, no product produced using a CFPS platform has achieved FDA approval, following disappointing Phase II/III results for Sutro’s antibody-drug conjugate (ADC) candidate [112]. Despite this, promising results from a clinical trial on a Vaxcyte 31-serotype conjugate pneumococcal vaccine could lead to the first FDA-approved drug [113].

Even with the approval of a single product, it will be hard to convince an entire field to make the switch to an *in vitro* system, especially when thinking about the lack of adoption of microbial production platforms, despite their presence for many decades [114]. Pharmaceutical companies still prefer tried and tested methods of mammalian cells for therapeutic production, particularly with antibodies, although the mRNA vaccines have helped alleviate some of the apprehensions over new technologies. The biomanufacturing industry is locked into decades of mammalian-based protein expression infrastructure, and the lack of adoption of alternative platforms is in part due to the cost of new equipment and the difficulties in re-obtaining regulatory approval with a new platform [115].

Ultimately, the cost of CFPS remains a challenge. A successful first use case may ease apprehension around investment in an unproven technology. If the field focuses on responsive, on-demand production, a whole new avenue of decentralized manufacturing opens up. The advantages of creating a shelf-stable product can also be a unique selling point and should be leveraged in any suggested application. Decentralized microbial CFPS also has clear value for low- and middle-income regions where access to cold-chain logistics and centralized biomanufacturing capacity remains limited.

Should the field even manage to overcome the reticence of manufacturers, we are still a long way from personalized medicine. The public have shown apprehension for novel medicines; if we are truly going to make the change to personalized medicine, we need a way to highlight that the benefits outweigh the risks. If the ultimate goal is to consider personalized medicines that can be made at the bedside, what we have learnt from the mRNA vaccines and the mixed reception is that this will be a daunting step in adopting the mindset of being given a vial of lyophilized lysate that is an add-water-and-go medicine. Encouragingly, the field has a long time to overcome this, and first, we need to change the mind of industry to adopt this new technology with little impact on the public’s perception with no change in the end product.

Where microbial CFPS could fundamentally reshape biomanufacturing is not by replacing large-scale fermentation, but by enabling an entirely different operational model. CFPS excels where traditional systems struggle: producing proteins rapidly, locally and flexibly without the need for cell expansion, sterility-controlled fermentation suites or cold-chain logistics. The successes of companies such as Sutro Biopharma and Vaxcyte Ltd suggest that manufacturers are beginning to accept CFPS-derived intermediates and conjugation strategies, and although a wholly CFPS-manufactured therapeutic has yet to achieve approval, the trajectory is encouraging.

The disruptive potential lies in the ability to reposition manufacturing from centralized facilities to modular, decentralized, microbe-derived systems that can be stockpiled, mobilized and activated on demand. Microbial CFPS is particularly well suited to this kind of manufacturing because microbial systems combine scalability with a well-established industrial knowledge base. The next step is to move microbial CFPS from a research-focused technique to a manufacturing approach with clearly defined processes that meet regulatory expectations.

## What must the field do next?

**Fig. 3.**
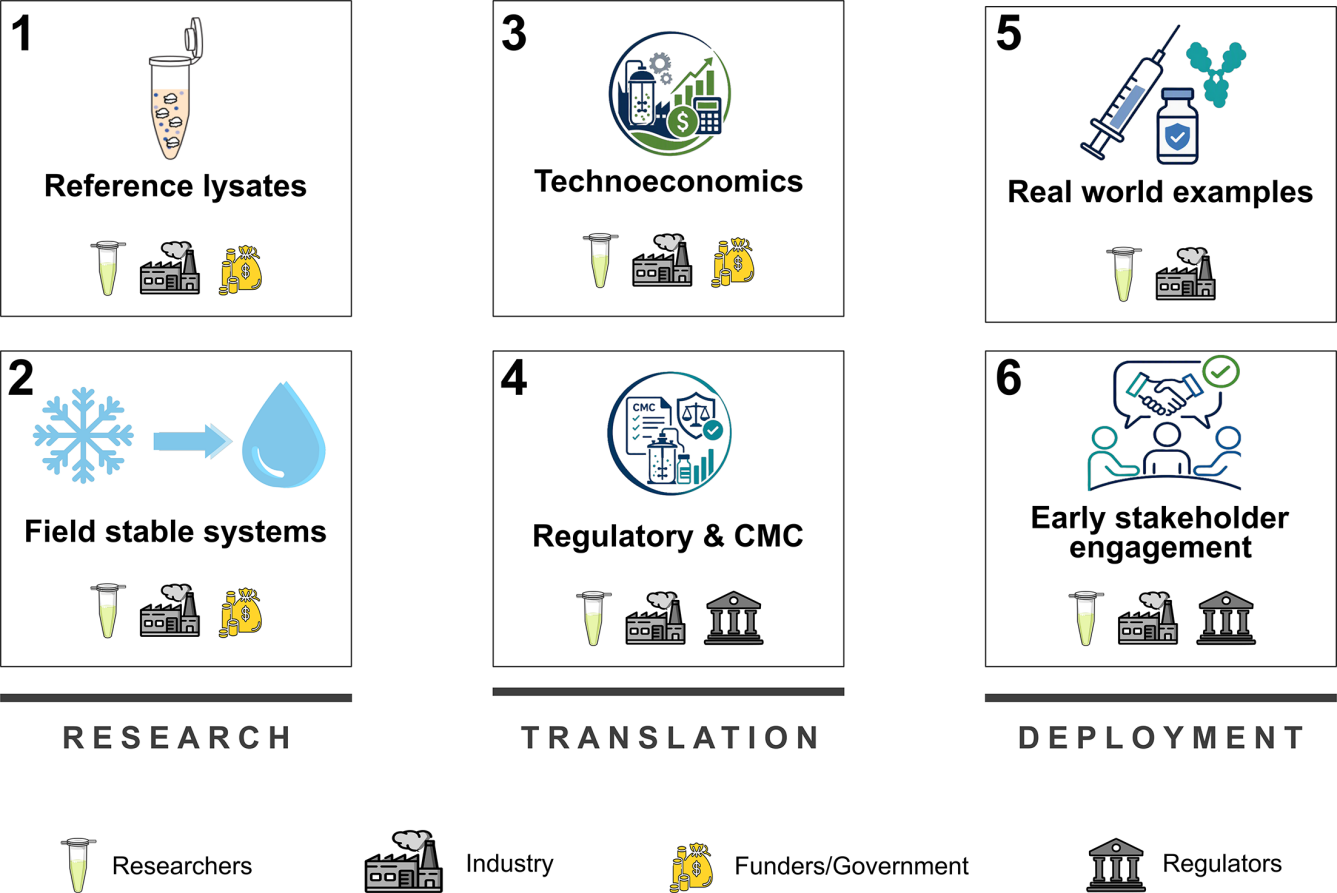
Roadmap for the translation of microbial CFPS into a biomanufacturing platform. Six key priorities are outlined alongside the primary stakeholders responsible for their implementation. These actions span extract standardization, formulation development, economic evaluation, regulatory alignment, demonstration of real-world utility and early cross-sector engagement. Progress will require coordinated efforts across academia, industry, regulatory bodies and funding organizations to enable deployment of CFPS technologies for decentralized manufacturing.

If microbial CFPS is to move from a research lab tool to a manufacturing technology, the field must adopt a co-ordinated approach (Fig. 3). Step-by-step optimization of microbial lysates will not be able to accomplish the overarching goals needed to create a biomanufacturing environment. Instead, standardization with benchmarking will be necessary to ensure that the field of microbial CFPS can justify the necessary risk associated with the adoption of the new technology.

### Establish microbial reference lysates with defined performance criteria

At minimum, the field requires well-characterized lysates, potentially covering a range of organisms to cover different microbial lysate attributes, such as *E. coli*, *K. phaffii*, a Gram-positive species (e.g. *Bacillus*) and one thermophile. Standard metrics of translation activity, endotoxin content (where applicable) and acceptable batch-to-batch variation are necessary for an honest assessment of productivity. This will add credibility to a new technology and provide confidence in the capability of this field and will be an essential aspect for regulatory purposes.

### Develop fully lyophilized, field-stable CFPS formulations

Shelf-stable, co-lyophilized systems that integrate lysate, energy reagents, salts and cofactors will be important for decentralized manufacturing. Achieving multi-month stability at room temperature (or longer if considering astropharmacy objectives) and validated performance after excessive temperature fluctuations should be considered critical milestones.

### Establish a technoeconomic foundation for microbial CFPS adoption

Industrial uptake requires predictable economics, yet there are only a handful of rigorous technoeconomic evaluations for CFPS [116, 117]. Both models specifically look at either antibody drug conjugates or monoclonal antibodies, in either *E. coli* or CHO lysates. The community must produce a wider variety of technoeconomic analysis models that capture full process costs: extract production, reagents, energy systems, lyophilization, quality control, labour and equipment load in varying lysates that could be commercially viable. These models should explicitly benchmark microbial CFPS against microbial fermentation for different product classes and volumes. Such analyses will reveal where CFPS is already competitive (e.g., small-batch, rapid-response manufacturing) but also to map where cost reductions will have the greatest return.

### Produce a CFPS-specific Chemistry, Manufacturing and Controls (CMC) and regulatory guidance framework

Industrial adoption requires a roadmap indicating what regulators will ask for. In collaboration with the US FDA, Sutro Biopharma set out to develop reference materials to improve regulatory standards [111]; however, no further information has yet been disseminated. While similar CFPS-specific initiatives have not yet been reported from European or UK regulatory bodies, there has been increasing regulatory attention toward decentralized and point-of-care manufacturing approaches [7]. These frameworks, particularly emerging UK guidance, demonstrate a growing acceptance of non-traditional production paradigms but have not yet explicitly addressed CFPS. As such, CFPS platforms are currently expected to be evaluated within existing biologics and Good Manufacturing Practice (GMP) frameworks, requiring developers to justify lysate-based production approaches on a case-by-case basis. The production of a bioindustrial manufacturing readiness level framework shows what the next steps could look like if applied to CFPS industrial adoption [118]. It will require endeavours such as these to assemble a CFPS CMC template outlining extract release criteria, contamination controls, in-process checks and equivalence arguments for lysate-based production.

### Provide a practical, real-world example

Microbial CFPS will gain credibility once it is shown to work for a real application, although efforts are well underway to achieve this.

### Engage early with industry and regulatory partners

To ensure adoption, manufacturers should be involved during technology development rather than after the fact. This will help align microbial CFPS with industrial expectations and regulatory realities in the attempt to de-risk the adoption of a new technology.

Collectively, these steps offer a pathway for microbial CFPS to mature into a robust, validated and widely trusted manufacturing platform. While the field has made significant improvements on a fundamental basis, to ensure implementation, it will be necessary to think beyond laboratory scale and consider regulatory elements and standards necessary to ensure industrial translation.

## Conclusion

The coming decade will determine whether microbial CFPS evolves into a biomanufacturing platform or remains primarily a laboratory technique. The scientific foundations are already strong; microbial lysates continue to improve in yield, and real-world examples are approaching regulatory review. The growing diversity of microbial hosts also broadens the likelihood that cell-free approaches can support a range of products rather than a single niche.

Manufacturers’ hesitancy is understandable; without a proven product and case study of success, there is substantial risk of implementation. At present, the field lacks validated extract standards, reproducible workflows, transparent costings and CFPS-specific regulatory guidelines, making it harder to evaluate risk or plan investment. The challenge, therefore, is not to generate yet more isolated improvements in extract yield or reaction composition, but to establish these benchmarks to allow for direct comparisons to microbial fermentation. This research has the potential to highlight not only the true cost of manufacturing with microbial CFPS but also the limitations that need to be addressed.

At the same time, we must remember that public perception and regulatory confidence will play a part in shaping decentralized or point-of-care manufacturing just as strongly as scientific innovation. While we are a long way from personalized medicine, the field is attempting to move in this direction, and if microbial CFPS is going to contribute to personalized therapeutics, we must consider biosafety, responsible use and broader scientific trust that will ensure microbial CFPS moves beyond the laboratory and contributes meaningfully to decentralized bioproduction.

## Abbreviations

AAV: adeno-associated virus
ADC: antibody-drug conjugate
AI: artificial intelligence
CFPS: cell-free protein synthesis
CHO: Chinese hamster ovary
CMC: Chemistry, Manufacturing and Controls
FDA: Food and Drug Administration
GMP: Good Manufacturing Practices
ML: machine learning
NTPs: nucleoside triphosphates
OSTs: oligosaccharyltransferases
PTMs: post-translational modifications
SARS-CoV-2: severe acute respiratory syndrome coronavirus 2
VLP: virus-like particle
